# Dental Hygiene

**Published:** 1889-02

**Authors:** R. H. Fitch

**Affiliations:** Pekin, Illinois.


ARTICLE IV.

### DENTAL HYGIENE.

BY DR. R. H. FITCH, PEKIN, ILLINOIS.

[Read before the Central Illinois Dental Society.]

In his great work on "American Nervousness", Dr. Beard says in substance that nervousness, which he has previously described as "nervelessness" or nerve exhaustion, is the reason for the early decay of the teeth; that Americans are the most nervous nation in the world, and have the worst teeth of any nation, which is the reason that American dentists are the best dentists in the world.

This statement of Dr. Beard is confirmed by the reports of the school inspectors of Paris, who find that the scholars in the public schools suffer from an accelerated decay of the teeth from the date of their entrance. Since my attention has been called to this phase of this subject, I have noted the general fact that among individuals great mental anxiety, as well as long fevers, caused especial sensitiveness of the teeth. It does not matter from what cause this nervousness proceeds, the effect is constant. I have noted this effect on the teeth among the students of our graded public schools, and especially in the case of those who were trying to do two years' work in one.

This American system of mental gormandizing, called education, is a constant menace to the most careful work of the dentist. The mental dyspepsia which follows this overloading of the brain deranges all the functions of nutrition alike. In addition to the terrible strain on the nerve forces from the regular routine of school life, there are so-called amusements and recreations—may God forgive us for lying—viz: children's exhibitions, parties, balls, May dances, etc., etc., by which the nerve tension is kept at the highest pitch; from which, indeed, the children early learn the old lesson, viz: "This world would be very agreeable if it were not for its pleasures." I once saw a French novel entitled "Memoirs of an Old Man of Twenty-five," but it seems that these children were always old men and old women. While the prophets seemed to miss the mark sometimes, they "hit it fair" when they said the child should die a thousand years old.

As these matters are the result of our social and educational systems, they are beyond our control, and we can only palliate, then, so far as the teeth are concerned by "patience and plastics." If by the use of gutta-percha and oxyphosphate of zinc the decay can be arrested till the "sweet girl graduates" the tooth tissues may become more dense while she is teaching or waiting for a husband.

If the foregoing reasoning is correct, then all hygiene is dental hygiene, and every thing which aids the general physical system is of advantage to the dental organs. And I wish to make some suggestions on the subject of general hygiene, believing that unless these are fully mastered our work will not reach the full measure of a reasonable success.

When Ingersoll was asked by the clergyman what he would have made different from the order of things if he had been the Creator, he promptly answered he would have made health catching instead of disease. There is no doubt that much health could be caught with less labor than boys spend in catching fish, or politicians in catching votes, and first to avoid "colds," catarrhs, congestions, Bright's disease, etc., etc., take care of the skin. In an old book is reported a conversation between two high authorities, as follows: "And Satan answered the Lord and said, skin for skin, yea, all that a man hath will he give for his life." And the succeeding experiments on old Job fully verified their judgment of importance of a healthy cuticle.

The value of salt baths as a stimulant to the healthy action of the skin has been recognized from the earliest ages. They can be taken thrice a week, or even daily, to advantage. Under this treatment catarrhs become a thing of the past—a "putrid reminiscence," as Story would say. Are congestions of the internal organs possible with the skin in healthful action? How can Bright's disease establish itself with the skin in perfect condition? And this bath is very easily taken. One-half cup of salt, three quarts of water, a sponge, one or two coarse towels, and a room only moderately warm; to be followed by dumb-bell exercise or even free gymnastics.

Now, if a man once becomes addicted to dumb-bells he will find it a very fascinating habit. It may even become like the "ruling passion strong in death," as with the great Forrest, who was found stark and cold with a heavy bell in each hand. A few minutes' exercise with the dumb-bells is followed by a sense of exhilaration and renewed life more charming than the potent influence of old wine. The quickened circulation of the blood, the expansion of the lungs, the elasticity and tension of the muscles, the clearing of the brain, the brightening of the eye, all indicate a re-creation of the vital force. It is as near being "born again" as falls to the lot of ordinary sinners.

Not wishing to bore you with a long paper, let me commend this subject to your earnest thought and kindly criticism.—*Dental Review.*

